# Community- and government-managed marine protected areas increase fish size, biomass and potential value

**DOI:** 10.1371/journal.pone.0182342

**Published:** 2017-08-14

**Authors:** Angelica A. D. Chirico, Timothy R. McClanahan, Johan S. Eklöf

**Affiliations:** 1 Department of Ecology, Environment and Plant Sciences (DEEP), Stockholm University, Stockholm, Sweden; 2 Marine Programs, Wildlife Conservation Society, Bronx, New York, United States of America; University of California Santa Cruz, UNITED STATES

## Abstract

Government-managed marine protected areas (MPAs) can restore small fish stocks, but have been heavily criticized for excluding resource users and creating conflicts. A promising but less studied alternative are community-managed MPAs, where resource users are more involved in MPA design, implementation and enforcement. Here we evaluated effects of government- and community-managed MPAs on the density, size and biomass of seagrass- and coral reef-associated fish, using field surveys in Kenyan coastal lagoons. We also assessed protection effects on the potential monetary value of fish; a variable that increases non-linearly with fish body mass and is particularly important from a fishery perspective. We found that two recently established community MPAs (< 1 km^2^ in size, ≤ 5 years of protection) harbored larger fish and greater total fish biomass than two fished (open access) areas, in both seagrass beds and coral reefs. As expected, protection effects were considerably stronger in the older and larger government MPAs. Importantly, across management and habitat types, the protection effect on the potential monetary value of the fish was much stronger than the effects on fish biomass and size (6.7 vs. 2.6 and 1.3 times higher value in community MPAs than in fished areas, respectively). This strong effect on potential value was partly explained by presence of larger (and therefore more valuable) individual fish, and partly by higher densities of high-value taxa (e.g. rabbitfish). In summary, we show that i) small and recently established community-managed MPAs can, just like larger and older government-managed MPAs, play an important role for local conservation of high-value fish, and that ii) these effects are equally strong in coral reefs as in seagrass beds; an important habitat too rarely included in formal management. Consequently, community-managed MPAs could benefit both coral reef and seagrass ecosystems and provide spillover of valuable fish to nearby fisheries.

## Introduction

Government-managed no-take marine protected areas (hereafter 'government MPAs') are an effective management tool when it comes to conserving local natural resources, including critical habitats, biodiversity and overharvested fish stocks [1–4]. However, human coastal populations, particularly those in development nations, rely on near-shore fish stocks for food, income and livelihoods [5–8]. Therefore, implementation of no-take areas can result in at least a temporary loss of income, biological knowledge and social capital [9], and ultimately cause or exacerbate conflicts [10–12]. A promising alternative to government MPAs are community MPAs (sometimes referred to as ‘community fisheries’ closures’ or ‘community-based reserves’), where local communities participate in or lead area-based management, for example through construction and enforcement of MPAs [9, 13]. Community MPAs have been shown to increase the biomass and size of coral reef-associated fish [e.g. 14, 15–18] and density of some target fish families [e.g. 19], even though not to the same extent as older and often larger government MPAs [20]. However, the extent to which community MPAs protect fish in other habitats than coral reefs is largely unknown.

In this study, we investigated effects of government and community MPAs on fish communities in shallow seagrass beds and coral reefs along the southern coast of Kenya, East Africa. In contrast to coral reefs, seagrass beds have only more recently been acknowledged as important providers of ecosystem services [5, 6, 21, 22], including fisheries [23, 24]. Even though many seagrass fish species are less sedentary and territorial than coral reef fish, seagrass cover affects fish communities both on the local [25] and regional scale [e.g. 26]. As a consequence, tropical seagrass beds are often preferred as fishing grounds, and seagrass-associated herbivorous fish can make up >50% of fish catches [27, 28]. Despite the pivotal role of seagrass, most tropical zone research and management still focuses on coral reefs [29, 30]. So far, very few studies have investigated effects of MPAs on seagrass-associated fish, but these indicate that there are positive effects on fish density, size and biomass [28, 31–33].

**Fig 1:**
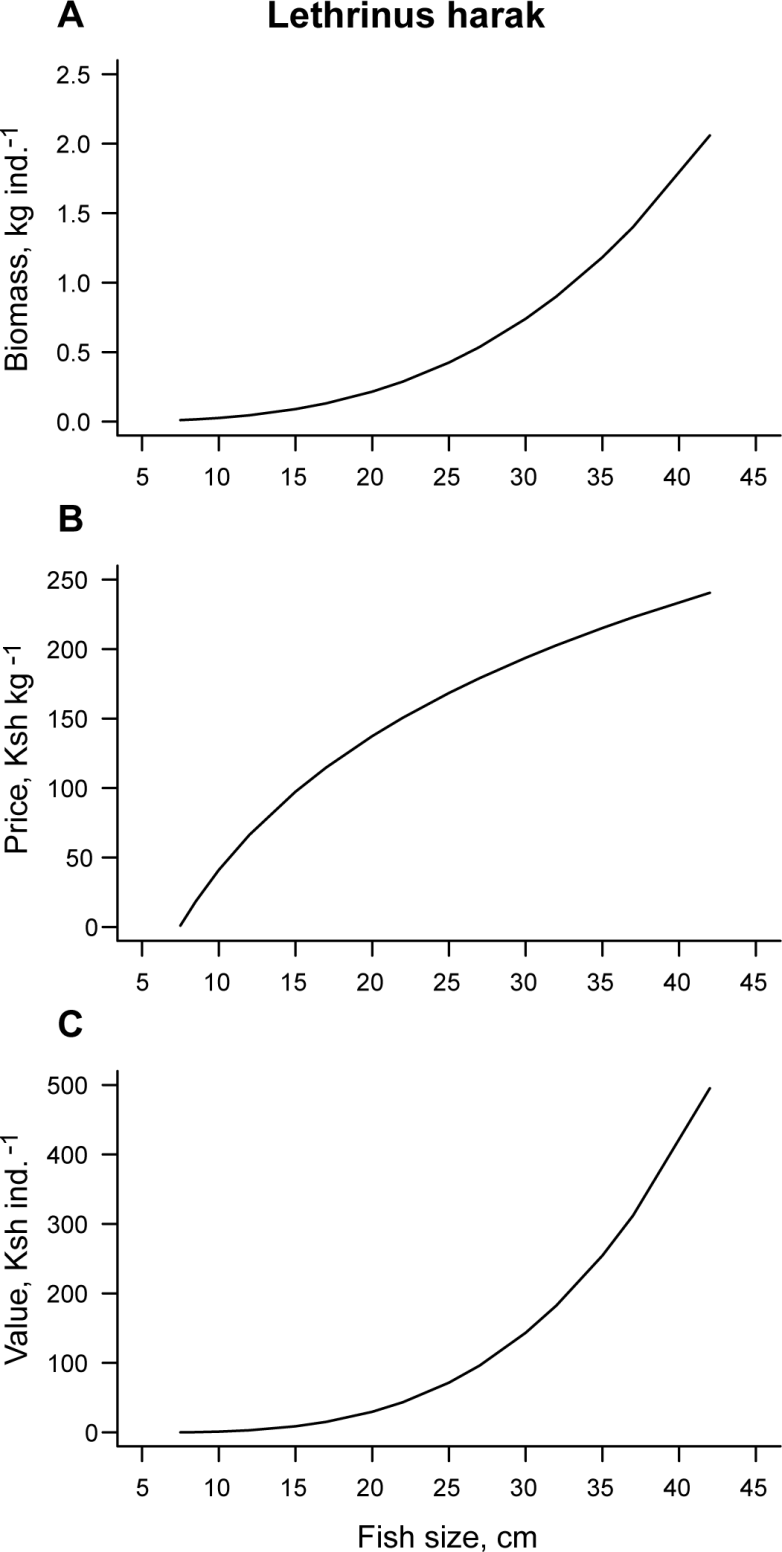
**Effect of individual fish size (standard length) on fish A) biomass, B) price per kilogram and C) potential monetary value.** The example shown is the species *Lethrinus harak* (Lethrinidae). A) Effect of individual size on biomass per individual (kg), calculated using species-specific size-weight relationship from FishBase [41]. B) Logarithmic effect of individual size on price per kg fish (in Kenyan shilling, Ksh), based on size-price relationships for scavengers in the study area [39]. C) As a consequence of the two relationships above, there is a steeply increasing non-linear effect of fish size on potential monetary value per fish.

Most studies examining MPA effects on fish communities use a few standard variables like fish density, size, biomass and community composition [e.g. 34, 35]. Positive effects on these variables are important because they indicate recovery from fishing, that in turn can generate spillover of fish (or larvae) to nearby fisheries [1, 36]. However, from a fishers´ perspective, effects on the potential monetary value of protected fish stocks, or the spillover they generate, is also an important variable because fish value ultimately affects income [37]. So far, very few studies have assessed effects of MPAs on monetary value of tropical fish stocks [28, 38]. Importantly, the market value of an individual fish is influenced not only by its biomass (which scales non-linearly with fish length: Fig 1A). Previous studies from the study area show that as fish grow in size, they move from low- to high-prized markets, e.g. from sales to households to restaurants and hotels [39, 40], and fish value per kg increases with fish body length (Fig 1B). Consequently, the market value per individual increase more steeply with fish size than what fish biomass does (Fig 1A vs. 1C). In addition, changes in fish species composition with protection to a higher dominance of high-value fishery species should increase the total value of the fish assemblage per unit area. In summary, we therefore predict that protection from fishing has a stronger positive effect on the monetary value of fish stocks than on standard metrics like fish density, size and biomass, at least during the initial phase of recovery.

Against this background, we surveyed fish communities in seagrass and coral reef areas and compared the effects of community MPAs on fish density, size and biomass, with the effects of larger and older government MPAs, using fished (open access) areas as reference. Using data from a 12-year fish market survey [39], we also estimated effects on the fish potential monetary value per unit area. Finally, we also assessed to what extent the presence and cover of habitat-forming organisms–here, reef-forming corals and seagrasses—influenced fish communities. We hypothesized that i) community MPAs positively affect fish communities in both seagrass and coral reef habitats, but not to the same extent as older government MPAs [20], ii) the effect of protection is stronger on potential monetary value of fish than on fish density, size and biomass (since large fish are disproportionally more valuable than small fish), and iii) the % cover of hard corals has a stronger effect on fish communities than seagrass cover, because coral reef fish are usually more sedentary and dependent on local habitat characteristics [42–44].

## Methods

### Study area

The Kenyan coastline (approximately 600 km) borders the Western Indian Ocean and is characterized by inter- and subtidal lagoons dominated by subtidal seagrass beds and coral reefs. A majority of the households depend on fishing for their income and daily food requirements [6]. At the same time, commercial fishes and their habitats are threatened by intense fishing and habitat destruction [45]. Since the 1960s, four government MPAs have been established in Kenya to protect natural resources and generate tourism revenues [30, 45]. Over the last decade, a number of coastal fishing communities have in collaboration with non-governmental organizations started creating community MPAs (Swahili: *tengefus*) to protect local fish stocks from overfishing [15].

### Survey design

**Fig 2:**
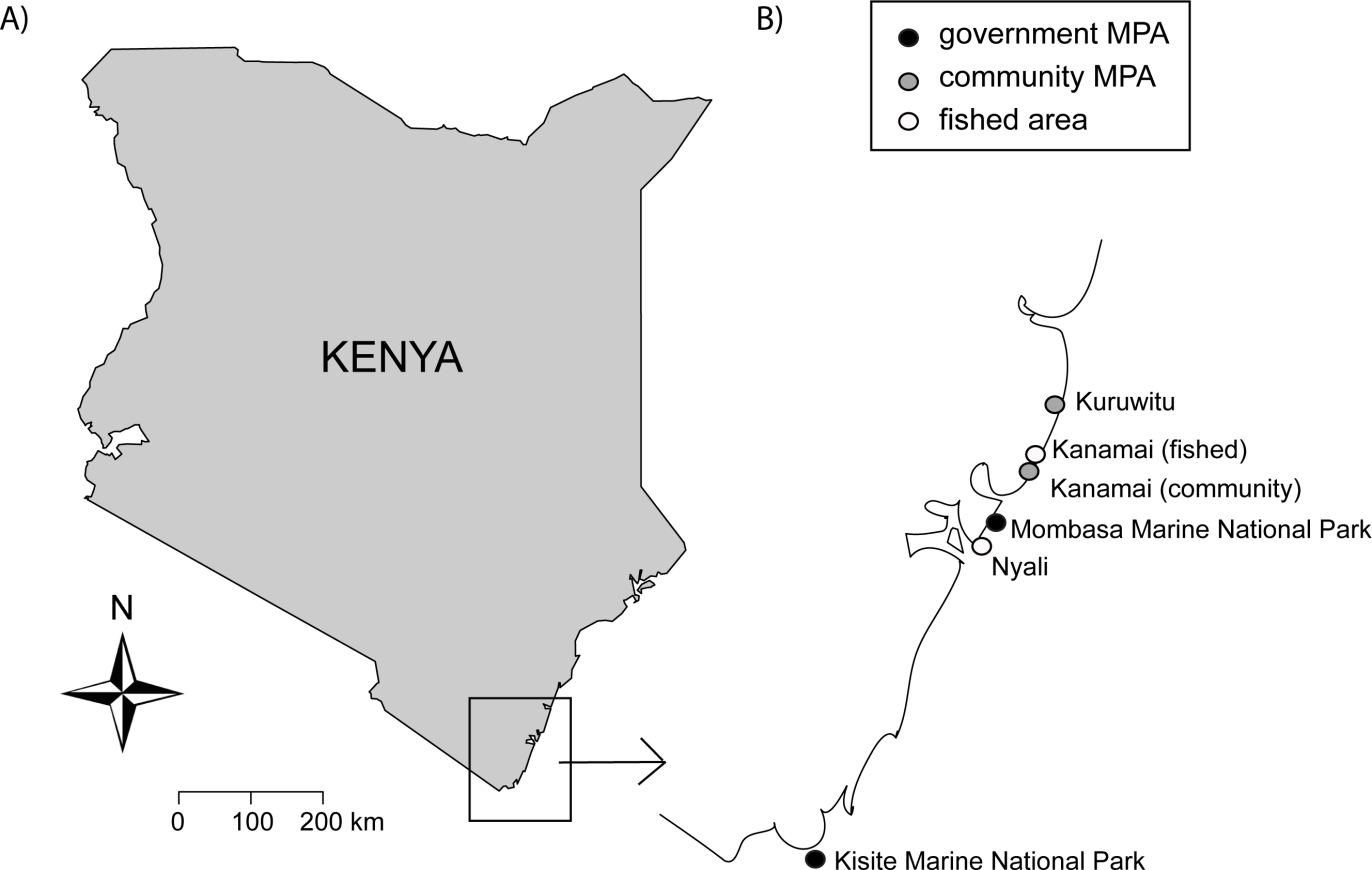
Map of study region. A) Map of Kenya (land in grey), noting the position of the study area in the rectangle. B) The southern Kenyan coastline, marking the positions of the six study areas with black, grey and white dots, respectively.

In November-December 2011 we surveyed fish communities and habitat characteristics in six areas along the southern Kenyan coast; two community MPAs (Kuruwitu and Kanamai), two government MPAs (Mombasa and Kisite Marine National Parks) and two fished (open access) areas (Nyali and Kanamai) (Fig 2, Table 1). The areas were selected as they i) represent one of the three types of management (government MPA, community MPA, fished area), ii) have roughly comparable coral reef and seagrass communities in terms of benthic cover, and iii) together span a wide range in number of years since closure ('age' of MPA). All areas except Kisite (which is located offshore near Kisite Island) were situated in shallow (<5 m depth) coastal lagoons protected by a fringing reef. Kuruwitu and Kanamai community MPAs have been protected from fishing since 2006 and early 2011, respectively, whereas Kisite and Mombasa Marine National Parks were established in 1978 and 1991, respectively [46, 47] (Table 1).

**Table 1:** Description of the six study areas.

Site name	Management type	Year established	Time since closure (years)*	Size of closure (km^2^)*	Proximity to human settlements (km)*
Nyali	fished	–	0	–	0.2
Kanamai	fished	–	0	–	Seagrass: 0.6 Coral: 0.9
Kanamai	community MPA	2011	1	0.22	Seagrass: 0.6 Coral: 0.9
Kuruwitu	community MPA	2006	5	0.40	Seagrass: 0.2 Coral: 0.4
Mombasa	government MPA	1991	20	6.00	1.0
Kisite	government MPA	1978	33	28.00	8.8

*: Note the strong correlations between time since closure and a) size of closure (r = 0.99, *P* < 0.001) and b) proximity to human settlements (r = 0.78, *P* < 0.001).

Due to the lack of data on fish communities before the community and government MPAs were implemented, we used a space-for-time substitution design to assess the effects of community and government MPAs; a common approach in MPA effect studies [see e.g. 3, 48 for a similar approach]. Surveys were conducted in two habitats located in central and shallow parts of the lagoons (< 2.5 m at low tide); i) seagrass beds dominated by *Thalassia hemprichii*, one of the most abundant and habitat-forming seagrasses in Kenya [49], and ii) coral reefs dominated by reef-forming scleractinian corals and macroalgae. All surveys were conducted within replicated 5×5 m (25 m^2^) point transects (n = 7–9 per habitat type and site, N = 92). Point transects are commonly used for fish surveys in seagrass beds [50–53] because they increase the chance of finding fish hiding in the complex seagrass canopy, as compared to regular line transects. Point counts have in some investigations been shown to yield higher fish densities than line transects [54, but see 55 and, 56]. Consequently, our estimates of absolute fish density, biomass and monetary value per unit area may not be directly comparable to those from line transects. The point transects were placed >30 m apart and >200 m from MPA borders, to avoid edge effects. To assess the effect of the cover of habitat-forming seagrasses and corals on fish communities, transects were placed in a stratified manner so that the 7–9 transects within each habitat type together covered as wide of a range in cover of seagrass or coral cover as possible within each site.

### Fish surveys

Point-counts were conducted by a single snorkeler (AC) during mid-neap tides between 09:00 and 15:00. First, the 5×5 m quadrate was marked with a rope tied to wooden sticks driven into the bottom and left for at least 20 minutes to minimize disturbance on the fish. Second, all diurnally active, non-cryptic individual fishes >3 cm (standard length) observed within the quadrate were visually identified and size estimated for 15 minutes. Fishes were identified to species [57], except for some species of surgeonfish [*Acanthurinae* other sp.], wrasse [Labridae other sp.], emperor [*Lethrinus* other sp.] and damselfish [Pomacentridae other sp.], and all species of the family scorpionfish [Scorpaenidae sp.] and rabbitfish [*Siganus* sp.], that were identified to genus or sometimes family (S1 Table). The observer was positioned at the shoreward corner of each transect and moved as little as possible to avoid disturbing fish. All fishes that swam in and out of the transect area were recorded for twelve minutes and care was taken not to double-count individuals moving in and out of the quadrat more than once. The remaining three minutes were spent searching for individuals hiding close to the bottom [following 50, 53]. Each fish was classified into one of the following size (cm) categories; 3–5, 6–9, 10–14, 15–19, 20–24, 25–29, 30–34 and 35–39. Individuals ≥40 cm and with slender body shape (i.e. trumpetfish [Aulostomidae], needlefish [Belonidae], moray eels [Muraenidae] and snake eels [Ophichthidae]) were estimated to the nearest cm [following 58].

### Benthic community surveys

The bottom cover (nearest 5%) of seagrasses (primarily *Thalassia hemprichii*, but pooling all species) and hard corals (pooling all species, including the hydrozoan *Millepora* and anthozoan *Tubipora* sp.), was estimated within a 0.5 m^2^ quadrat randomly placed five times within each 25 m^2^ point transect. The mean seagrass vs. coral cover was calculated per transect, and used as a replicate in the statistical analyses (see below).

### Estimations of fish biomass

To estimate total fish biomass (kg wet mass per transect), individual fish weight was estimated using species-specific length-weight relationships from FishBase [41]. When multiple length-weight relationships existed for a taxa, priority was given to metrics based on greatest number of replicates and geographical region closest to the study area [following 16]. For fish <40 cm in length, the mean length of its size category was used to calculate weight (so that, for example, a fish from category 15–19 cm was given the length of 17 cm). For large (≥ 40 cm) and slender-bodied species, the exact length was used. In cases when standard length-to-weight relationships were missing, our standard length (mean of the size category) was first transformed to fork- or total length using length-length data from FishBase [41]. For individuals that could not be identified to species level, mean values for the most common species from the same family within the study area were used. Finally, we summed up the total fish biomass (pooling all individuals) per transect.

### Estimations of potential monetary value of fish

To calculate the potential monetary value of the surveyed fish communities (Kenyan Shilling per 25 m^2^), we combined the size and biomass estimations (see above) with size:value relationships for five value groups, based on Kenyan fish market data; i) scavengers (including emperors [Lethrinidae], snappers [Lutjanidae] and grunts [Haemulidae]), ii) goatfish (Mullidae), iii) rabbitfish (Siganidae), iv) parrotfish (Scaridae) and v) 'rest of catch' (low-value fish commonly sold on markets but not readily categorized into species or groups) [see 39]. Fish size (standard length) and corresponding market value (Kenyan shilling per kilogram) were extracted from McClanahan (39). Separate size-value relationships for each fish group were estimated by comparing the fit of linear, logarithmic and exponential linear models to the market data (for best-fitting models, see S1 Fig). Total monetary value per 25 m^2^ was then calculated based on these size-value relationships. All individuals from the smallest size category (3–5 cm) were excluded prior to analysis, because they possess almost no market value [39] and including them in the equations (which were based on larger fish) would have generated negative values. Consequently, our analysis ignores the fact that these smaller fish may still have a subsistence value (see Discussion). In addition, individuals from the families trumpetfish, needlefish, moray eels and snake eels were also excluded from the value calculations, since fish with slender body overestimate the actual value. Moreover, four poisonous species (*Diodon liturosus*, *Arothron meleagris*, *A*. *nigropunctatus* and *Canthigaster valentini*) [41] were also excluded.

### Statistical analyses

#### Effects on fish density, size, biomass and potential market value

The effects of management type, habitat type and seagrass vs. coral cover on fish density, size, biomass and potential monetary value, were assessed using general linear mixed models in R version 3.3.1 [59]. The models included the predictors 'management type' (fixed with three levels: fished, community MPA, government MPA), 'habitat type' (fixed with two levels: seagrass beds vs. coral reefs), ‘foundation species cover’ (fixed, continuous variable; expressed as seagrass cover in the seagrass habitats, and coral cover in the coral habitats) and their interactions, while accounting for differences between sites (random factor), using the *nlme* package [60]. As 'Kanamai community MPA' and 'Kanamai fished' were situated very close to each other, they were treated as coming from the same site (resulting in 5 sites in total). Prior to analyses, assumptions of normally distributed errors were checked using residual plots, and homogeneity of variances by plotting residuals against fitted values. We also tested for multicollinearity by assessing the variance inflation factor (VIF), and checked for outliers using Cleveland dot-plots and boxplots. When necessary, response variables were log or square root transformed. Starting with the full model (including all predictors and their interactions), we then used model selection based on maximum likelihood and the Akaike´s Information Criterion corrected for small samples (AICc) [61] to compare the fit of all possible models. A ΔAICc > 2 units was regarded to signal a model with superior fit. We then identified the most parsimonious models (that included only significant terms at α = 0.05, using restricted maximum likelihood). To identify which of the three management types that differed when the factor 'management type' was significant, we conducted multiple comparison tests (Tukey HSD; α = 0.05) using the *multcomp* package [62]. Differences between management types and habitats were displayed using box plots.

Since recovery of fish communities from fishing can take several decades [7, 46, 63, 64], we then ran a second set of similar models that included 'time since closure' (fixed, continuous variable; 0–33 years) instead of 'management type’. By accounting for differences in time since closure between sites of the same management type (see Table 1), these analyses should be statistically more powerful. Effects of time since closure and cover of foundation species in the two habitats were displayed using partial regression plots using the *visreg* package for R [65].

We also tried to assess if the size of individuals from the most common species were affected by MPAs or time since closure, but the very low densities per species in some sites resulted in too low power to perform meaningful analyses (results not shown).

Another factor that in theory could influence both seagrass and coral reef fish communities, and potentially influence our assessment of MPA effects, is the proximity (distance, in km) to human settlements (hereafter ‘proximity’); a coarse indicator of human impacts [66]. In our data set, this factor was strongly correlated with time since closure (r = 0.78, *P* < 0.001; Table 1), primarily because Kisite–the MPA protected the longest (33 years)–is situated nearly 9 km from nearest human settlement, while the rest of the sites (protected for 20 years or less) are situated <1 km from human settlements. To avoid issues of multicollinearity but still be able to statistically assess the relative influence of proximity in relation to time since closure, we first excluded the obvious outlier; the data from Kisite. This reduced the strength of the positive correlation (r = 0.67), but also reduced the overall sample size (from 92 to 76). Combined with adding one more predictor (proximity) to an already complex statistical model, the full model may easily be overfitted to the data. Consequently, we chose to assess the relatively influence of proximity to human settlements only on total fish biomass; a variable that incorporates responses in both fish density, size, and species composition. Model selection resulted in three models within 2 AICc units: i) intercept + time of protection + habitat (AICc = 77.0), ii) intercept + time of protection * habitat (AICc = 78.58), and iii) intercept + time of protection + habitat + proximity (AICc = 78.6). The first model was not only simplest (fewest parameters) but had >2 times higher AICc weight (0.26 vs. 0.11). Moreover, adding ‘proximity’ to the model did not improve model fit (ANOVA, *P* = 0.38). Consequently, the variability in fish biomass appears to more influenced by the time of protection from fishing and differences between habitats, than of the proximity to human settlements. Consequently, we chose not to include ‘proximity’ as a factor in the rest of the analyses.

#### Effects on fish value group composition

Protection and habitat effects on fish monetary value could in theory be caused by changes in fish size and/or density, but also by changes in community composition based on fish value groups. Therefore, we also tested effects of management type (fixed, three levels), habitat type (fixed, two levels), foundation species cover (fixed, continuous variable) and site (random factor with 6 levels) on fish community composition based on densities of the five fish value groups (see above), using a mixed model permutated analysis of variance (PERMANOVA). The reason for here using six levels of the random factor was that PERMANOVA requires that all levels of the fixed factor are nested within different levels of the random factors. Fish density was fourth root-transformed prior to analysis to decrease the impact of the most abundant groups, and p-values ('P-perm') were obtained using 9999 permutations of Bray-Curtis similarity indices under unrestricted permutations of raw data; an approach suitable for smaller sample sizes [67]. Preliminary analyses using the adonis() function from the *vegan* package [68] (with ‘site’ as a strata) indicated that variability in the fish community was mainly driven by management, habitat types and sites, and not by foundation species cover. Therefore, we chose to i) exclude foundation species cover (which had no significant effects in the adonis analyses) and ii) analyze the data using Primer v 6.1.15 [following 69], which provides more flexibility in terms of accounting for the random variability and perform post-hoc tests. Significant PERMANOVA results were further explored using the SIMPER routine, which indicates which fish groups that contribute the most to the detected differences between factor levels.

To assess whether 'time since closure' (continuous variable), habitat and cover of foundation species affected fish group composition, we also performed an *adonis* analysis (with site as a strata). To minimize skewness and avoid outliers, the predictor variable 'time since closure' was square root-transformed prior to analyses. P-values were also here obtained using 9999 permutations of Bray-Curtis similarity indices. Significant factors were explored using Draftsman plots of Pearson linear correlations (r) between predictor and response variables.

## Results

In total, 2176 fishes from 111 diurnally active, non-cryptic species or higher taxa were observed (for species list see S1 Table). The species belonged to 31 families commonly found in seagrass and coral reef habitats in the Western Indian Ocean region [57, 70]. All data is supplied in our supporting information (S1 and S2 Dataset).

### Total fish density

**Fig 3:**
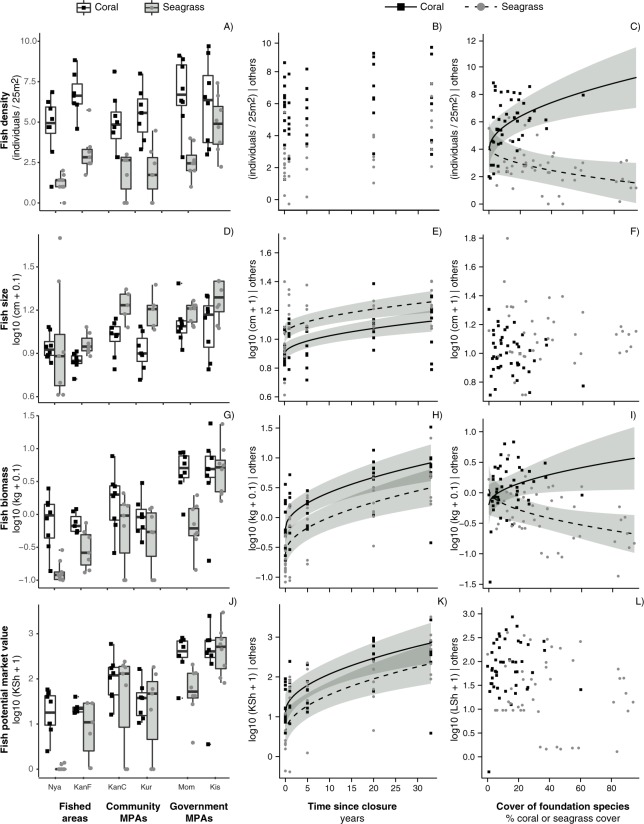
Effects of MPAs on fish density, size, biomass and value in seagrass beds and coral reefs. First column: effects of fisheries management (fished areas, community MPAs, government MPAs) in coral and seagrass habitats on fish A) density (no. ind), D) size (mean standard length, cm), G) biomass (kg), and J) potential value (Kenyan shilling) per 25 m^2^ (median ± 75^th^ and 25^th^ quantile and 95% CI, n = 7–9). Site abbreviations: Nya = Nyali, KaF = Kanamai fished, KaC = Kanamai community MPA, Kur = Kuruwitu, Mom = Mombasa and Kis = Kisite. Second column: effects of time since closure (years) in coral and seagrass habitats on fish B) density, E) size, H) biomass, and K) potential value (best-fitting partial regression ± 95% CI, n = 46 per habitat). Third column: effects of cover of foundation species (coral cover in coral sites, seagrass cover in seagrass sites) on fish C) density, F) size, I) biomass, and L) potential value (best-fitting partial regression ± 95% CI, n = 46 per habitat).

Total fish density differed between management types, with ca. 2 times more fish individuals in government than community MPAs (Table 2, Fig 3A). There was no difference in density between the MPAs and the fished areas. Moreover, fish density was 3.9 times higher on coral reefs than in seagrass beds (Table 2, Fig 3A). There was also an effect of foundation species cover that differed between the two habitats (interaction effects; Table 2): in the coral reef sites fish density increased with coral cover, whereas in the seagrass sites fish density instead decreased with coral cover (Fig 3C).

**Table 2:** Effects of management type, habitat type and foundation species cover on univariate fish metrics. Summary of minimal adequate linear models assessing the effects of i) management type (fixed, 3 levels: fished, community MPA, government MPA) ii) habitat type (fixed, 2 levels: seagrass bed, coral reef), iii) foundation species cover, and iv) their interactions, on 1) fish density (individuals per 25 m^2^), 2) fish size (standard length, cm), 3) total fish biomass (kg per 25 m^2^) and 4) total potential monetary value (Kenyan shilling per 25 m^2^).

	F	P*	Tukey HSD post-hoc test
**1. Density**			
Management type	3.51	**0.035**	Government MPA > community MPA
Habitat type (H)	98.19	**<0.001**	coral > seagrass
Foundation species (F)	0.28	0.59	
F × H	16.64	**<0.001**	Increase with coral cover, decrease with seagrass cover
**2. Size**			
Management type	15.13	**<0.001**	fished < (community MPA = government MPA)
Habitat type	15.36	**<0.001**	seagrass > coral
**3. Biomass**			
Management type	15.86	**<0.001**	fished < community MPA < government MPA
Habitat type (H)	30.87	**<0.001**	coral > seagrass
Foundation species (F)	0.87	0.37	
F × H	12.50	**<0.001**	Increase with coral cover, decrease with seagrass cover
**4. Potential value**			
Management type	15.37	**<0.001**	fished < community MPA < government MPA
Habitat type	15.09	**<0.001**	coral > seagrass

*Numbers in bold indicate statistically significant values (*P* < 0.05).

The analyses including ‘time since closure’ instead of ‘management type’ showed that the simplest model did not include an effect of time since closure (Table 3, Fig 3B). However, total density was higher in coral reefs than in seagrass beds, and increased with coral cover in the coral sites, but decreased with seagrass cover in the seagrass sites (Table 3, Fig 3C).

**Table 3:** Effects of time since closure, habitat type and foundation species cover on univariate fish metrics. Summary of minimal adequate linear models assessing the effects of i) time since closure (continuous variable, 0–33 years), ii) habitat type (fixed, 2 levels: seagrass bed, coral reef), iii) foundation species cover, and iv) their interactions, on fish 1) density (individuals / 25 m^2^), 2) size (standard length, cm), 3) total biomass (kg / 25 m^2^) and 4) total potential monetary value (Kenyan shilling / 25 m^2^).

	F	P*	Tukey HSD post-hoc test
**1. Density**			
Habitat type	94.06	**< 0.001**	coral > seagrass
Foundation species (F)	0.38	0.54	
F × H	17.30	**< 0.001**	Increase with coral cover, decrease with seagrass cover
**2. Size**			
Time since closure	25.07	**< 0.001**	
Habitat type	13.49	**< 0.001**	seagrass > coral
**3. Biomass**			
Time since closure	24.97	**< 0.001**	
Habitat type (H)	30.92	**< 0.001**	coral > seagrass
Foundation species (F)	0.35	**< 0.001**	
F × H	10.06	**< 0.001**	Increase with coral cover, decrease with seagrass cover
**4. Potential value**			
Time since closure	15.37	**< 0.001**	
Habitat type	15.09	**<0.001**	coral > seagrass

*Numbers in bold indicate statistically significant values (*P* < 0.05).

### Mean fish size

Fishes were on average 1.3 times larger in community MPAs and 1.6 times larger in government MPAs than in fished areas, respectively (Table 2, Fig 3D). Meanwhile, there was no difference in size between community and government MPAs (Table 2). Moreover, fish in seagrass beds were 1.5 times larger than fish on coral reefs (Table 2, Fig 3D); an effect largely attributed to higher densities of small damselfish (*Pomacentridae*) and wrasse (*Labridae*) on the coral reefs. Finally, fish size increased with time since closure in both coral and seagrass habitats (Table 3, Fig 3E). There were no effect of coral or seagrass cover on fish size (Tables 2 and 3, Fig 3F).

### Total fish biomass

Total fish biomass was 2.6 times greater in community MPAs and 10.8 times greater in government MPAs than in fished areas, respectively (Table 2, Fig 3G). Moreover, the biomass in the government MPAs was 4.1 times higher than in community MPAs (Table 2, Fig 3G). In terms of habitat differences, biomass was 1.9 times greater in coral reefs than in seagrass beds (Table 2, Fig 3G). There was also an effect of cover of foundation species, that differed between the two habitats (habitat × foundation species cover interaction; Table 2). In the coral reef sites fish biomass increased with coral cover, while in the seagrass beds, fish biomass decreased with seagrass cover (Fig 3I). Finally, fish biomass increased with time since closure in both habitats (Table 3, Fig 3H).

### Total potential monetary value of fish

Total potential monetary value of fish was 6.7 and 33.3 times higher in community MPAs and government MPAs than in fished areas, respectively, and was 4.9 times higher in government MPAs than in community MPAs (Table 2, Fig 3J). Fish value was 1.3 times higher on coral reefs than in seagrass beds (Table 2, Fig 3J). Finally, time since closure had a strong positive effect on fish value in both habitats (Table 3, Fig 3K). None of the best-fitting models included an effect of coral or seagrass cover (Table 2, Table 3; Fig 3L).

### Effects on fish community composition

Fish community composition based on densities of five value groups (scavengers, goatfish, rabbitfish, parrotfish and 'rest of catch') did not differ between management types, whereas habitat type and site affected fish group composition (Table 4). The value groups 'rest of catch' and parrotfish were more abundant on coral reefs (contributed to 61, 14 and 5% of the differences, respectively), and scavengers and rabbitfish were more abundant in seagrass beds (17 and 5% contribution, respectively). No interaction was found between management and habitat, but there was an interaction between site and habitat (Table 4).

**Table 4:** Effects of management type, time since closure and habitat type on fish community composition. Summaries of A) 3-factor PERMANOVA on effects of management type (3 levels; fished, community MPAs, government MPAs), habitat type (2 levels; coral reefs, seagrass beds) and site (random factor, 6 levels) and B) adonis analysis based on effects of time since closure in and habitat (seagrass vs. coral) on fish value group composition (density of scavengers, rabbitfish, goatfish, parrotfish and ‘rest of catch’).

**A. PERMANOVA**	**Pseudo F**	*P* (perm)*
Management type: M	1.07	0.44
Habitat type: H	11.46	**< 0.001**
Site (Management): S(M)	4.19	**< 0.001**
M × H	1.02	0.47
S(M) × H	2.65	**0.0053**
**B) adonis**	**Pseudo F**	P*
Time since closure	4.22	**<0.001**
Habitat	28.10	**<0.001**

*Numbers in bold indicate statistically significant values (*P* < 0.05).

The adonis analyses showed that time since closure changed the composition of value groups across habitat types (Table 4). On the coral reefs the three groups 'rest of catch', rabbitfish and scavengers correlated the strongest with time since closure (r = 0.39, 0.23 and 0.17, respectively). In the seagrass beds the three groups that correlated the strongest with time since closure were rabbitfish, 'rest of catch' and goatfish (r = 0.51, 0.43, 0.35, respectively). There was also a considerable difference in value group composition between the two habitat types: goatfish, parrotfish and ‘rest of catch’ were more common in the coral reefs, while rabbitfish and scavengers were more common in the seagrass beds.

## Discussion

Using field surveys along the Kenyan coastline we found that community MPAs, just as government MPAs, harbored larger sized fish and fish communities with higher biomass and much higher potential monetary value than fished reference areas. These results not only confirm earlier studies showing that recently established community MPAs can benefit coral reef fish biomass [14–18]; they also demonstrate that these effects occur in seagrass beds, which make up a considerable proportion of these tropical lagoons [71] but have received much less research and management focus than coral reefs. Previous studies have suggested that only a few years of protection may be enough for fished coral reef species to start to recover [e.g. 2, 72], even though the process may take considerably longer for large predators [73]. These results are supported by our study, suggesting effects of community MPAs protected for ≤5 years. MPA size, which can also positively affect fish communities [e.g. 63], differed greatly between the small community MPAs and large government MPAs in our study, and was positively correlated with MPA age (see Table 1). However, a large body of studies from Kenya, including >15 years of before-after surveys in Mombasa Marine National Park [46–48] show that time since closure has a strong, predictable effect on fish biomass, that closely track temporal patterns across other Kenyan MPAs of different ages [48]. A simple comparison of our results, which are based on a site-for-time comparison *between* fished areas and small community MPAs, and changes observed *within* large government MPAs over time, indicate similar temporal changes in fish biomass (S2 Table). Therefore, we suggest that the differences we find between community and government MPAs are primarily caused by the differences in time since closure, rather than MPA size. Combined with the fact that community MPAs often have higher acceptance among local communities than government MPAs [9, 74], our results suggest that community MPAs can be a promising alternative that help maintain and restore ecological values in coastal areas.

In both community- and government MPAs the protection effect on fish potential monetary value were stronger than the effects on regular monitoring variables like fish density, size and biomass. For example, community MPAs had on average three times higher fish biomass, but seven times higher potential monetary value, than fished areas. These results mirror those found in the Caribbean [38] and the Western Pacific [28], and were in our case explained by two mechanisms. First, protection increased the size of individual fish, which in turn has a stronger effect on fish market value than fish biomass [39, 40]. Second, there was a positive effect of time since closure on the density of high-value species (primarily rabbitfish; *Siganidae*). The disproportionally strong effect on fish market value could be important for several reasons. First, the rapid increase in fish value following the initiation of small community MPAs could provide incentive to implement community-based temporal closures, that once re-opened to fisheries can generate financial benefits to coastal communities [75]. Second, the apparently rapid increase in fish value within the community MPAs could with fish migrations generate ´spill over´ to fished areas and there benefit fisheries [1, 36], particularly since high-value rabbitfish (one of the groups increasing the fastest with time since closure) have been shown to migrate far from the MPAs where they were tagged [76]. One caveat to the strong effects on fish value is that we excluded small (≤ 5 cm) and non-market fish from the value analysis, while these fish may still have a subsistence value [28, 77]. Future studies should incorporate both market and non-market values of these fish communities, to provide a more complete view of MPA effects. Nevertheless, our results suggest that accounting for the disproportionally strong protection effects on fish monetary value may better capture the actual protection effects on these social-ecological systems. Third and final, not only fish market value but also many ecological processes/functions increase non-linearly with fish body size; e.g. grazing rates on macroalgae [78] and female reproductive output [79]. We therefore predict that size-dependent ecological functions also increase more rapidly with time since closure than total fish biomass and density. Consequently, other metrics incorporating non-linear effects of fish body size could be incorporated into future assessments of MPA effects.

Most studies investigating effects of MPAs have focused on hard bottom ecosystems like tropical coral reefs and temperate macroalgal reefs [29, 30]. Here, we show that effects of MPAs in seagrass beds were strikingly similar to effects in nearby coral reefs. To our knowledge, this is the first study to demonstrate MPA effects on seagrass fish communities in Sub-Saharan Africa and the Western Indian Ocean, and one of the very few studies to assess MPA effects in seagrass beds globally [28, 31–33]. Since seagrass beds support a wide range of ecosystem services [5, 6, 21], one being fisheries [23, 24], our results are important from several perspectives. On the one hand, we show that small-scale, artisanal fisheries can reduce fish density, size and biomass, and alter the composition of seagrass fish communities. Given the importance of seagrass fish for food security in many tropical areas, there is a clear need for improved seagrass fisheries management. On the other hand, we show that small, recently established community MPAs positively affect the size, biomass and value of seagrass fish, and therefore could play an important role as a management tool to sustain seagrass ecosystems services.

Regardless of the type of management or time since closure, the studied coral reefs had higher fish density, biomass and potential monetary value of fish per unit area than seagrass bed [see also 51, 53]. Moreover, in the reef habitats fish biomass and density increased with local coral cover, whereas in the seagrass habitats, fish density and biomass decreased weakly with increasing seagrass cover. Even though these contrasting effects were not studied in more detail, there are several potential explanations to the patterns. First, coral reefs are inhabited primarily by small, sedentary and relatively territorial species like damselfish, who are strongly dependent on the quality of the local habitat [44]. Meanwhile, most tropical seagrass fish (e.g. snappers, emperors, rabbitfish and parrotfish) are highly mobile and less linked to the local habitat [76]. Second, it is possible that high cover (i.e. a very dense shoot canopy) of the relatively short and structurally simple seagrass *Thalassia hemprichii* actually reduces the role of seagrass as a shelter, by leaving too few spaces for fish to hide [80]. The important role of seagrasses as fish habitats has been studied primarily in temperate areas, but also in the tropics, where seagrass habitat complexity can play an important role. A recent study conducted around the nearby island Zanzibar (Tanzania) found a clear, positive effect of the cover of the larger, structurally more complex seagrass species *Thalassodendron ciliatum* on seagrass fish communities [25]. Moreover, presence and areal cover of seagrass beds has been shown to be highly important for fish biomass not only in seagrass beds [5, 6, 21], but also in nearby coral reefs [26, 51]. Consequently, even though changes in local coral cover are likely to have a stronger effects on the local fish community, loss of seagrass cover–especially of large, habitat-forming species–is also likely to affect seagrass-associated fish and fisheries negatively [81].

## Conclusions

Our study suggests that small and recently established community MPAs (< 1 km^2^, ≤ 5 years of protection), can increase fish size and total biomass, just like government MPAs (> 6 km^2^; ≥ 20 years of protection). Moreover, MPA effects on the potential monetary value of fish were stronger than effects on fish density, size and biomass. Therefore, community MPAs may rapidly benefit local fish stocks, and could (at least in theory) also benefit spillover of mobile, high-value individuals (e.g. large rabbitfish) [76] to nearby fisheries, just as government MPAs. In addition, both community and government MPAs benefitted seagrass fish communities in similar ways as coral reef fish; an important finding given the few studies assessing effects of MPAs in seagrass beds globally. Consequently, MPAs can benefit seagrass-associated fishes and, potentially, seagrass fisheries. Since seagrass beds support many near-shore fisheries in developing countries [20, 27, 28, 82] but are threatened on a global scale [22], both community- and government-managed MPAs are likely to play an important role for conservation of seagrass associated fishes and their associated values.

## Supporting information

S1 Fig Relationships between fish size and market value. Linear relationships between fish size (standard length, cm) and market value (price in Kenyan shilling, Ksh/kg) for five value groups.
(DOCX)

S1 Dataset Data used for univariate analyses. (XLSX)

S2 Dataset Data used for multivariate analyses. (XLSX)

S1 Table List of fish taxa found during the study in the six study areas in Kenya. (DOCX)

S2 Table **Comparison of MPA effects based on A) time series within MPAs and B) site-for-time surveys between MPAs.** Relative increase in mean total fish biomass based on time series analysis within large government MPAs (data from McClanahan and Graham 2005) and site-for-time survey (this study) show similar results and suggest that time since closure is an important factor, regardless of size of closure or type of management.
(DOCX)
