# Supplementary material for: Community- and government-managed marine protected areas increase fish size, biomass and potential value
Source: PLoS One. 2017 Aug 14;12(8):e0182342. doi: 10.1371/journal.pone.0182342 (PMC5555630; doi:10.1371/journal.pone.0182342)
Supplement: S1 Fig — Linear relationships between fish size (standard length, cm) and market value (price in Kenyan shilling, Ksh/kg) for five value groups. (DOCX) [file pone.0182342.s001.docx]

**Figure S1. Relationships between fish size and market value**

Method: to estimate the effect of fish size (standard length, cm) on market value (price per kg in in Kenyan shilling), we first extracted data on size vs. value for five value groups - scavengers (emperors, snappers and grunts), goatfish, rabbitfish, parrotfish and ‘rest of catch‘ (category of low-value fish commonly sold on markets but not readily categorized into species or groups) - from [1]. We then fitted linear, logarithmic and exponential linear regression models to each data set, and report the best-fit relationship (based on AIC). The relationships were later combined with fish biomass estimates, to calculate the value per individual fish, and the total value of all fish per transect.

**

**

Figure S1. Linear relationships between fish size (standard length, cm) and market value (price in Kenyan shilling, Ksh/kg) for five value groups; A) scavengers (emperors, snappers and grunts), B) goatfish, C) rabbitfish, D) parrotfish and E) ‘rest of catch‘ (group of low-value species commonly sold on markets but not readily categorized into species or groups). Based on data from [1].

[1] McClanahan TR (2010) Effects of fisheries closures and gear restrictions on fishing income in a Kenyan coral reef. Conservation Biology 24: 1519-1528.
