# Supplementary material for: Community- and government-managed marine protected areas increase fish size, biomass and potential value
Source: PLoS One. 2017 Aug 14;12(8):e0182342. doi: 10.1371/journal.pone.0182342 (PMC5555630; doi:10.1371/journal.pone.0182342)
Supplement: S1 Table — (DOCX) [file pone.0182342.s004.docx]

**Table S1.** **List of fish taxa found during the study in the six study areas in Kenya.**

| **Family** | **Fish species or group** | **Family** | **Fish species or group** |
| --- | --- | --- | --- |
| Acanthuridae | *Acanthurus dussumieri* | Labridae | *Cheilinus trilobatus* |
| Acanthuridae | *Acanthurus leucosternon* | Labridae | *Cheilio inermis* |
| Acanthuridae | *Acanthurus nigricauda* | Labridae | *Coris aygula* |
| Acanthuridae | *Acanthurus tennentii* | Labridae | *Coris caudimacula* |
| Acanthuridae | *Acanthurus triostegus* | Labridae | *Coris formosa* |
| Acanthuridae | *Ctenochaetus strigosus* | Labridae | *Gomphosus caeruleus* |
| Acanthuridae | *Naso annulatus* | Labridae | *Halichoeres hortulanus* |
| Acanthuridae | *Naso elegans* | Labridae | *Halichoeres nebulosus* |
| Acanthuridae | *Naso unicornis* | Labridae | *Halichoeres scapularis* |
| Acanthuridae | *Paracanthurus hepatus* | Labridae | *Hemigymnus melapterus* |
| Acanthuridae | *Zebrasoma scopas* | Labridae | *Labroides bicolor* |
| Acanthuridae | *Acanthurinae* other sp. | Labridae | *Labroides dimidiatus* |
| Apogonidae | *Cheilodipterus quinquelineatus* | Labridae | *Novaculichthys taeniourus* |
| Aulostomidae | *Aulostomus chinensis* | Labridae | *Oxycheilinus bimaculatus* |
| Balistidae | *Balistapus undulatus* | Labridae | *Stethojulis albovittata* |
| Balistidae | *Pseudobalistes fuscus* | Labridae | *Stethojulis strigiventer* |
| Balistidae | *Rhinecanthus aculeatus* | Labridae | *Thalassoma hardwicke* |
| Balistidae | *Rhinecanthus rectangulus* | Labridae | *Thalassoma hebraicum* |
| Balistidae | *Sufflamen chrysopterum* | Labridae | *Thalassoma lunare* |
| Belonidae | *Strongylura incisa* | Labridae | Labridae other sp. |
| Caesionidae | *Caesio lunaris* | Lethrinidae | *Lethrinus harak* |
| Carangidae | *Carangoides ferdau* | Lethrinidae | *Lethrinus mahsena* |
| Carangidae | *Caranx melampygus* | Lethrinidae | *Lethrinus obsoletus* |
| Carangidae | *Caranx sexfasciatus* | Lethrinidae | *Lethrinus* other sp. |
| Carangidae | *Scomberoides lysan* | Lutjanidae | *Aprion virescens* |
| Chaetodontidae | *Chaetodon auriga* | Lutjanidae | *Lutjanus bohar* |
| Chaetodontidae | *Chaetodon guttatissimus* | Lutjanidae | *Lutjanus fulviflamma* |
| Chaetodontidae | *Chaetodon kleinii* | Monacanthidae | *Cantherhines pardalis* |
| Chaetodontidae | *Chaetodon lunula* | Mullidae | *Parupeneus barberinus* |
| Chaetodontidae | *Chaetodon trifasciatus* | Mullidae | *Parupeneus ciliatus* |
| Chaetodontidae | *Chaetodon vagabundus* | Mullidae | *Parupeneus macronemus* |
| Cirrhitidae | *Paracirrhites forsteri* | Muraenidae | *Echidna nebulosa* |
| Diodontidae | *Diodon liturosus* | Nemipteridae | *Scolopsis ghanam* |
| Haemulidae | *Plectorhinchus flavomaculatus* | Ophichthidae | *Myrichthys maculosus* |
| Haemulidae | *Plectorhinchus gaterinus* | Ostraciidae | *Lactoria fornasini* |
| Holocentridae | *Myripristis murdjan* | Platycephalidae | *Papilloculiceps longiceps* |
| Holocentridae | *Neoniphon sammara* | Pomacanthidae | *Centropyge multispinis* |
| Holocentridae | *Sargocentron diadema* | Pomacanthidae | *Pomacanthus imperator* |
| Kyphosidae | *Kyphosus cinerascens* | Pomacanthidae | *Pomacanthus semicirculatus* |
| Labridae | *Anampses caeruleopunctatus* | Pomacentridae | *Abudefduf sexfasciatus* |
| Labridae | *Bodianus axillaris* | Pomacentridae | *Abudefduf sparoides* |
| Labridae | *Cheilinus chlorourus* | Pomacentridae | *Abudefduf vaigiensis* |

| **Family** | **Fish species or group** |
| --- | --- |
| Pomacentridae | *Amphiprion allardi* |
| Pomacentridae | *Chromis viridis* |
| Pomacentridae | *Dascyllus aruanus* |
| Pomacentridae | *Dascyllus trimaculatus* |
| Pomacentridae | Pomacentridae other sp. |
| Scaridae | *Calotomus carolinus* |
| Scaridae | *Calotomus spinidens* |
| Scaridae | *Chlorurus sordidus* |
| Scaridae | *Hipposcarus harid* |
| Scaridae | *Leptoscarus vaigiensis* |
| Scaridae | *Scarus falcipinnis* |
| Scaridae | *Scarus frenatus* |
| Scaridae | *Scarus ghobban* |
| Scaridae | *Scarus psittacus* |
| Scaridae | *Scarus rubroviolaceus* |
| Scaridae | *Scarus tricolor* |
| Scorpaenidae | Scorpaenidae sp. |
| Serranidae | *Cephalopholis argus* |
| Serranidae | *Cephalopholis boenak* |
| Serranidae | *Epinephelus merra* |
| Siganidae | *Siganus* sp. |
| Tetraodontidae | *Arothron meleagris* |
| Tetraodontidae | *Arothron nigropunctatus* |
| Tetraodontidae | *Canthigaster bennetti* |
| Tetraodontidae | *Canthigaster solandri* |
| Tetraodontidae | *Canthigaster valentini* |
| Zanclidae | *Zanclus cornutus* |
