# Supplementary material for: Community- and government-managed marine protected areas increase fish size, biomass and potential value
Source: PLoS One. 2017 Aug 14;12(8):e0182342. doi: 10.1371/journal.pone.0182342 (PMC5555630; doi:10.1371/journal.pone.0182342)
Supplement: S2 Table — Comparison of MPA effects based on A) time series within MPAs and B) site-for-time surveys between MPAs. Relative increase in mean total fish biomass based on time series analysis within large government MPAs (data from McClanahan and Graham 2005) and site-for-time survey (this study) show similar results and suggest that time since closure is an important factor, regardless of size of closure or type of management. (DOCX) [file pone.0182342.s005.docx]

**Table S2. Comparison of MPA effects based on A) time series within MPAs and B) site-for-time surveys between MPAs.** Relative increase in mean total fish biomass (based on coral reef fish > 10 cm) based on A) time series analysis within large government MPAs (data from McClanahan and Graham 2005) and B) site-for-time survey comparing fished areas and small community MPAs (this study). Even though our study is based on a site-for-time replacement method, the similar increase in both studies (135 vs. 166% increase in fish biomass over 3.5 years) suggest that time since closure is an important factor, regardless of size of closure or type of management.

| **Study type** | **Management type** | **Biomass, ha^-1^ , 0 years of protection** | **Biomass, ha^-1^ , 3.5 years of protection** | **% increase from 0 to 3.5 years of protection** |
| --- | --- | --- | --- | --- |
| **A. Time series**  McClanahan and Graham 2005* | government MPA | 334 | 570 | 339/252 = 1.345 ≈ 135 % |
| **B. Site-for-time survey**  Our study (Chirico *et al.* 2016) | community MPA | 214 | 570 | 356/214 = 1.664 ≈ 166 % |

* Figure 2C page 244 in McClanahan TR, Graham NAJ (2005) Recovery trajectories of coral reef fish assemblages within Kenyan marine protected areas. Marine Ecol Prog Ser 294: 241-248.
